# *Alarmin*’ Immunologists: IL-33 as a Putative Target for Modulating T Cell-Dependent Responses

**DOI:** 10.3389/fimmu.2015.00232

**Published:** 2015-06-02

**Authors:** Tania Gajardo Carrasco, Rodrigo A. Morales, Francisco Pérez, Claudia Terraza, Luz Yáñez, Mauricio Campos-Mora, Karina Pino-Lagos

**Affiliations:** ^1^Centro de Investigación Biomédica, Facultad de Medicina, Universidad de Los Andes, Santiago, Chile; ^2^Facultad de Ciencias, Universidad de Chile, Santiago, Chile; ^3^Programa Disciplinario de Inmunología, Instituto de Ciencias Biomédicas, Facultad de Medicina, Universidad de Chile, Santiago, Chile

**Keywords:** IL-33, T cells, tolerance, transplantation

## Abstract

IL-33 is a known member of the IL-1 cytokine superfamily classically named “atypical” due to its diverse functions. The receptor for this cytokine is the ST2 chain (or IL-1RL1), part of the IL-1R family, and the accessory chain IL-1R. ST2 can be found as both soluble and membrane-bound forms, property that explains, at least in part, its wide range of functions. IL-33 has increasingly gained our attention as a potential target to modulate immune responses. At the beginning, it was known as one of the participants during the development of allergic states and other Th2-mediated responses and it is now accepted that IL-33 contributes to Th1-driven pathologies as demonstrated in animal models of experimental autoimmune encephalomyelitis (EAE), collagen-induced arthritis, and trinitrobenzene sulfonic acid-induced experimental colitis, among others. Interestingly, current data are placing IL-33 as a novel regulator of immune tolerance by affecting regulatory T cells (Tregs); although the mechanism is not fully understood, it seems that dendritic cells and myeloid suppressor-derived cells may be cooperating in the generation and/or establishment of IL-33-mediated tolerance. Here, we review the most updated literature on IL-33, its role on T cell biology, and its impact in immune tolerance.

## Introduction

In 2005, IL-33 was first described as a member of the IL-1 family using computational sequence analysis, which revealed the existence of a β-trefoil-fold structure in its C-terminal domain, a characteristic feature of IL-1 family members such as IL-1β and IL-18 (1). In the same work, it was also found that IL-33 interacts with the IL-1 family receptor ST2, previously described as an orphan receptor expressed on Th2 cells and mast cells. When activated with IL-33, ST2 promotes Th2 cell responses over Th1 responses (1), and negatively regulates TLR–IL-1R receptor signaling (2).

**Figure 1:**
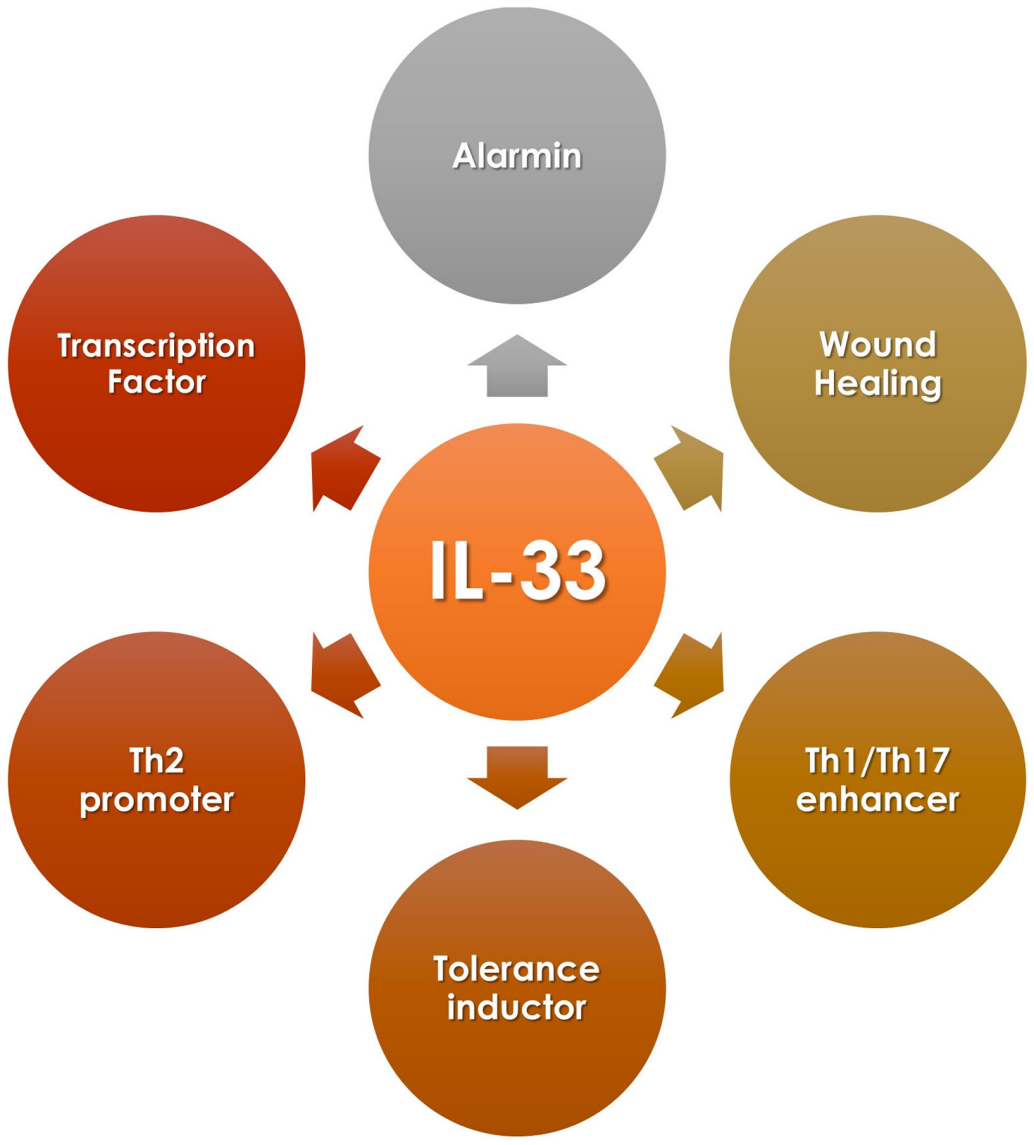
**IL-33 diversity of functions**.

IL-33 is known as an *alarmin* because of its high expression in endothelial and epithelial cells exposed to tissue damage or pathogen encounter, in addition to its accumulation in the nucleus of those cells, similar to other alarmin members of the IL-1 family such as HMGB1 and IL-1α (3). Therefore, upon injury or infection, IL-33 is released from the nucleus of endothelial cells to the extracellular space where it can signal and activate immune cells, such as mast cells, eosinophils, basophils, natural killer cells, and T cells (as many other functions described in this review) (Figure 1) (4).

Human IL-33 is a 30 kDa protein that shares 54% amino acid identity with its mouse homolog (5). Along its 270 amino acid sequence, we can describe a distinctive C-terminal IL-1-like cytokine domain of the IL-1 cytokine family and a central domain (1). IL-33 lacks a clear signal peptide, but it is synthesized with an N-terminal pro-peptide containing a nuclear localization domain (3, 6), which seems to be essential to IL-33 function *in vivo* because specific deletion of this nuclear domain (using a *knock-in* mouse model) results in a systemic, ST2-dependent, non-resolving lethal inflammation due to constitutive release of IL-33 into the circulation (7).

**Figure 2:**
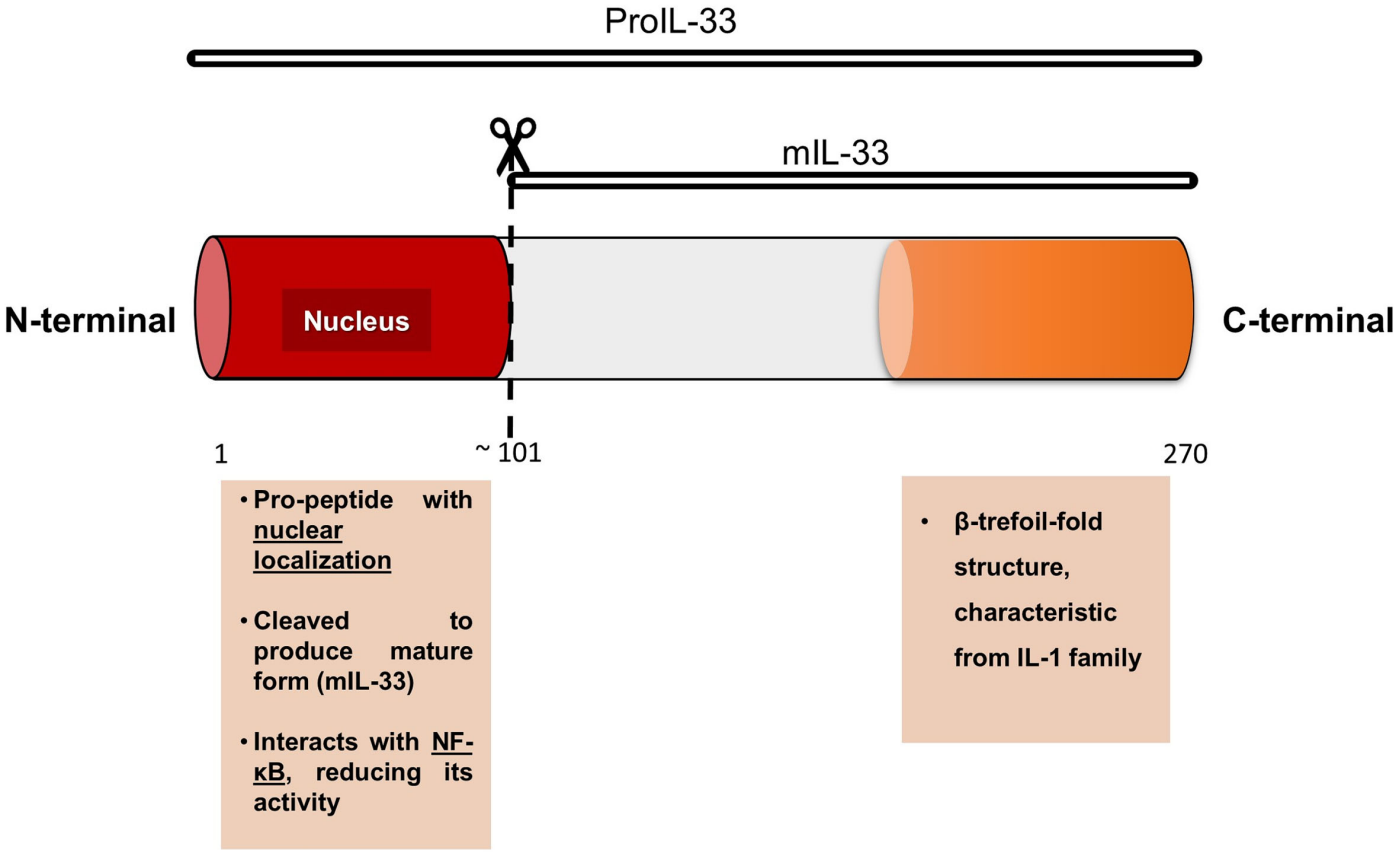
**Model of IL-33 protein, with its two principal domains and functions**.

As other members of the IL-1 family, IL-33 can suffer post-translational modifications resulting in two different forms: the full-length protein (proIL-33) and the processed or ‘mature’ form (mIL-33). mIL-33 is formed after proIL-33 cleavage in its N-terminal domain, but the cleavage sequence is different compared to other members of the IL-1 family, such as proIL-1β and IL-18 (8) (Figure 2). In the extracellular space, IL-33 binds to its membrane receptor ST2 activating the MyD88-dependent pathway, which is involved in cytokine secretion, cell activation, and differentiation (9). IL-33 can also bind to the soluble form (sST2) of its receptor, which corresponds to a decoy receptor that captures IL-33, avoiding or blocking intracellular signaling (10). Recently, it has been highlighted that the two forms of IL-33 (proIL-33 and mIL-33) are biologically active and they can trigger different immune responses (11).

The nuclear localization of IL-33 led to study a possible role in controlling gene expression, as discussed below.

## IL-33 as a Transcription Factor

As mentioned, IL-33 sequence does not contain a clear signal peptide, suggesting an (ancestral) intracellular localization. This observation suggests that IL-33 has a role as intracellular factor. Based on the similarities of IL-33 with other members of the IL-1 cytokine family, and the high-mobility group box-1 (HMGB1) protein, Carriere and colleagues studied the sequence, molecular, and cellular function of IL-33. In the first set of experiments, the group confirmed IL-33 nuclear localization, and interestingly, this location overlapped with high concentrations of DNA, suggesting that endogenous IL-33 binds to heterochromatin. This observation was confirmed using GFP-tagged IL-33 constructs, which, in addition, indicated that IL-33 binds to mitotic chromatin (12). Sequence and mutagenic experiments indicated that the N-terminal section of the protein is necessary for nuclear targeting, binding to heterochromatin and mitotic chromosomes, specifically the homodomain-like helix–turn–helix segment. Making the association that heterochromatin relates with unreachable or unexposed DNA, the investigators assayed IL-33 gene expression regulatory properties using a luciferase-based reporter system and showed that IL-33 acts as a repressor factor (12). In another report, it was described that endogenous IL-33 interacts physically with the transcription factor NF-κB. By performing co-immunoprecipitation experiments and microscopy, the investigators demonstrated that IL-33 binds to a free and activated NF-κB. In other words, IL-33 does not interact with κB (13). Supporting Carriere’s report, this group also found that IL-33 N-terminal sequence interacts with NF-κB, reducing its binding to DNA and repressing NF-κB transcription factor activity, as shown in EMSA and luciferase-based reporter experiments. Altogether, these reports indicate that endogenous IL-33 acts as a transcription factor (Figure 1).

## A Multifaceted Cytokine

### IL-33 and APCs

In addition to the role in the activation of the immune responses, IL-33 has also different roles in T cell polarization by modulating APCs. For example, IL-33 promotes the expansion of APCs, principally dendritic cells (DCs), by stimulating the secretion of basophils-derived GM-CSF (14). In a different study, Rank and colleagues found that DCs express high levels of intracellular ST2, and that the *in vitro* treatment of DCs with IL-33 induces the secretion of IL-6, upregulates the expression levels of MHC-II and CD86, and triggers the polarization of naïve T cells toward a Th2 phenotype (15). Similarly, Besnard and colleagues showed that IL-33 exposure induces the recruitment and activation of DCs in the lung, and their posterior migration to lymph nodes for antigen presentation in an allergic airway inflammation model (16). Alternatively, an indirect role of IL-33 on DCs was described by Duan and colleagues, who showed that IL-33 indirectly modulates intestinal DCs phenotype toward a tolerogenic profile given by the expression of CD103+CD11c+ (17). Supporting this potential tolerogenic effect, it was recently shown that IL-33 promotes IL-2 secretion by DCs, favoring the expansion of Foxp3+ Tregs cells (18).

### IL-33 and CD4+ T cells

A lot has been described about the abilities of IL-33 to modulate the immune response. One of our particular interests is the capacity to direct CD4+ T cells polarization to different phenotypes, with the purpose to manipulate immunity under pathogenic conditions, favoring a healthy state. In this line, we believe IL-33 is a good candidate to exploit, and here we review IL-33 effects on different T cells subsets (Table 1).

**Table 1:** **IL-33 effects in T cells**.

Cell type	Experimental model	Observations	Reference
CD4 Thl/Thl7	EAE	IL-33 blockade during MOG-dependent development reduces disease severity by decreasing IFN- γ and IL-17	(19)
		Treatment with exogenous IL-33 increases disease severity through IFN-γ and IL-17 secretion αlL-33 treatment improves symptoms	
	EAE	IL-33 treatment improves symptoms	(20)
	RA, CIA	IL-33 induces Th1 and Th17 responses	(21–23)
		ST2 blockade reduces severity by diminishing IFN-γ and IL-17 production		
	CIA, AIA	Presence of IL-33 did not affect development of the disease	(24)	
	OVA-induced allergic asthma	IL-33 induces the secretion of IL-6 and IL-1β from mast cells, promoting Th17 differentiation	(25)

CD4 Th2	Experimental atherosclerosis	IL-33 upregulates serum levels of Th2 cytokines and enriches for CD4+ST2+ T cells	(26)	
	Innate immunity	IL-33 acts on mast cells, basophils, eosinophils, type 2 innate lymphoid cells (ILC2), which promotes the secretion of Th2 cytokines, favoring Th2 polarization	(22, 27–29)

CD4 Tregs	Chronic colitis	Treatment with IL-33 reduces disease symptoms through a decrease on IFN-γ production, switching from a Th1 to a Th2 type response	(17, 30, 31)
		IL-33 affects directly Tregs in mice	
		IL-33 expands Tregs Foxp3+ST2+		
	EAE	IL-33-treated mice show an increase in Tregs and M2 macrophages	(20)
	Atherosclerosis	IL-33 prevents the development of atherosclerosis in Apo3^−/−^ mice	(26, 32)
		IL-33 treatment produces an increment in IL-4, IL-5, and IL-13 production and a decrease in IFN-γ levels	
		IL-33/ST2 axis is involved in the development of the disease		
	Transplantation	Heart-transplanted mice treated with IL-33 show increased graft survival	(33)	
		IL-33 treatment increases the number of CDllb + GRlint MDSC and Tregs, in addition to a less IL-17 production and elevated levels of IL-5, IL-13, and IL-10	

CD8		IL-33 is up-regulated in CD8+ T cells	(34)
		ST2 expression is T-bet-dependent	
		IL-33 plus IL-12 enhance CD8+ T cell effector phenotype	
	Lymphocytic choriomeningitis virus	IFN-γ, ST2, and IL-33 expressions were upregulated after infection	(35)
	Herpesvirus	IL-33 signaling is necessary for an effective CTL response	(35)
	Tumor	Tumor-derived IL-33 inhibits tumor growth and blocks the entrance of tumor-infiltrating lymphocytes (TILs)	(36)
		Mice-bearing IL-33-secreting tumor have more activated and differentiated CD8+ T cells, which is related to T-bet and Eomes expression	

### IL-33 on Th1/Th17

The context in which IL-33 is present influences directly the kind of immune response triggered. Thus, in certain cases, IL-33 can induce a Th1- or Th17-type response (Figure 1). These outcomes participate in the development and establishment of inflammatory responses that usually relate to diseases, such as autoimmunity, or to detrimental inflammatory responses such as graft-versus-host disease (GvHD) for the case of transplantation. Because of this, to understand the conditions when this cytokine would induce an inflammatory response over a tolerogenic one, it is essential when considering candidates for therapeutic interventions.

We believe that IL-33 versatility has contributed to the contradictory studies using animal models. For example, in the experimental autoimmune encephalomyelitis (EAE) model, some investigators have reported that IL-33 blockade during the developing stage of MOG-induced EAE reduces the severity of the disease, in part, due to the inhibition of MOG-dependent IL-17 and IFN-γ production. Unexpectedly, the treatment with exogenous IL-33 increased the severity of the disease by enhancing the production of these cytokines. Conversely, the treatment with an anti-IL-33 antibody reversed the symptoms and improved the state of the mice (19). In contrast with these findings, Jiang and colleagues observed an improvement in EAE mice after IL-33 treatment (20). When comparing both works, we identified differences that may contribute to obtain such contradictory results as the immunization (MOG peptide) site/location, timing, and peptide amount. Nevertheless, the kinetics and severity of the disease are equal in both cases. In addition, IL-33 treatment differs in both works; Li et al. injects the mice every other day, since day 0–18 with 50 μg/kg; while Jiang’s group treat the mice with 1 μg total from day 12 to 20 after immunization. These differences, especially in the treatment, can be related to the different results obtained, leading us to think that the effect given by IL-33 is time dependent and may affect different cell populations in each case. The IL-33 receptor ST2 can be expressed on astrocytes in the nervous system (37); thus, a potential impact of this cytokine on glial cells is very likely and may vary with the inflammatory state of them.

Similar to the EAE model, conflicting data are also reported for rheumatoid arthritis (RA). For example, some studies indicate that IL-33 is prejudicial for this disease because it induces a Th17-type response, which aggravates the symptoms (21, 22). In collagen-induced arthritis (CIA), it has been described that IL-33 has similar effects, developing a strong autoimmune response mediated by Th17 cells (22). According to these data, the blockade of ST2 helps to diminish the severity of CIA through a decrease in the production of IFN-γ and IL-17 (21, 23). However, other authors state that the role of IL-33 in this specific disease is not relevant. For example, Talabot-Ayer and colleagues showed in models of CIA and antigen-induced arthritis (AIA) that IL-33 knock-out mice develop the disease despite the lack of IL-33. They conclude that IL-33 does not play an essential role for the development of the disease, but it may contribute to the inflammatory environment (24).

Other disease linked to IL-33 is asthma. When IL-33 expression is deregulated, Th2 cells, mast cells, basophils, and others cells get activated triggering the expression of cytokines and chemokines that characterizes the disease (38). However, in a model of OVA-induced allergic asthma, the authors described a predominant Th17-type response, which is induced by the activation of ST2/IL-33 signaling on mast cells. The authors observed that mast cells from IL-33-treated mice produced IL-1β and IL-6, collaborating with Th17 cells development (25).

These few examples show us the pleiotropic abilities of this cytokine, which can direct CD4+ T cells differentiation to Th1 and Th17, causing and participating in different inflammatory states and diseases.

### IL-33 on Th2

As mentioned earlier, IL-33 can also modulate Th2 type responses (Figure 1) in addition to its effect on Th1 cells. *In vitro* and *in vivo* data suggest that IL-33 indirectly promotes Th2 responses functioning as a chemoattractant for this type of cells (39, 40). Other studies demonstrated that IL-33 could directly act on CD4+ST2+ Th2 cells promoting the secretion of IL-4, IL-5, and IL-13 (40–42). IL-33 has shown a protective role in the ApoE^−/−^ experimental atherosclerosis mice model, increasing serum levels of Th2-type cytokines, and enriching ST2+CD4+ T cells in lymph nodes (26). These observations indicate that IL-33 affects Th2 cell cytokine production in a TCR-independent fashion. Moreover, IL-33 promotes Th2-type responses by signaling on other immune cells rather than CD4+ T cells. In this case, it has been reported that IL-33 induces mast cells activation, maturation, and cytokine production such as IL-5, IL-6, IL-8, and IL-13 (22, 27, 43). In addition to this, IL-33 can also modulate Th2 immunity by acting on basophils, eosinophils, and more recently described, on newly identified type-2 innate lymphoid cells (ILC2), which includes nuocytes, natural helper cells, and innate helper type-2 cells. On these cells, IL-33 promotes IL-5 and IL-13 secretion (29, 42, 43–45).

Taking all together, there are sufficient indications supporting the role of IL-33, either directly or indirectly, in Th2-mediated immune responses.

### IL-33 on Tregs

In addition to the evidence mentioned above, it is reported that IL-33 can also expand and promote Tregs accumulation, among other effects, helping to establish tolerance (Figure 1). Different animal models support this evidence, from autoimmunity to organ transplantation settings.

For the case of autoimmunity, one of the models studied is chronic colitis. In this approach, the investigators observed that mice treated with exogenous IL-33 manifested a decrease in disease symptoms, which was associated to a down-regulation on IFN-γ production. This intervention on the cytokine environment led to a shift of the T helper response, from Th1 to Th2, reducing inflammation in the intestine (30). This work demonstrated the capacity of IL-33 to modulate immune responses in this organ, but it does not imply necessarily a direct role for Tregs. However, in another model of colitis, which is induced by trinitrobenzene sulfonic acid (TNBS), the protective role of IL-33 was also observed. In this case, the authors evaluated the contribution of Tregs by depleting these cells in IL-33-treated diseased animals, obtaining a loss in protection compared to non-depleted animals or, in other words, Tregs contribute to IL-33-mediated protection (17). One could argue that IL-33 may or may not be acting on CD4+ T cells because ST2 is expressed by a wide range of cells; nevertheless, a Treg-dependent response is seen demonstrating that CD4+ T cells can rapidly react (directly or not) to IL-33 stimulation. Supporting these findings, Schiering and colleagues described that IL-33 treatment expands Tregs, increasing Foxp3 and ST2 expression in the spleen of mice with ongoing colitis. In this report, IL-33 treatment did not disturb effector T cell function, but adoptive transfer experiments indicated that IL-33 acts on thymic Tregs (natural Tregs, nTregs) rendering a stable phenotype by “fixing” Foxp3 expression (therefore better suppressors) (31).

Since the expression of ST2 is widely distributed in cells of the nervous system, the role of IL-33 signaling has been also studied in the EAE model (46). In this experimental setting, IL-33 treatment incremented the frequency of Tregs and polarized macrophage phenotype toward M2 type in the spinal cord of EAE mice (20).

Furthermore, in atherosclerosis, a typical Th1-type pathology in which the frequency and function of Tregs are reduced, it was observed that IL-33 treatment of Apo3^−/−^ mice with a high-fat diet (model for this disease) prevented the development of atherosclerotic plaques, which is the first symptom of this disease. In addition, the authors obtained an increment on IL-4, IL-5, and IL-13 production, leading to a decrease in IFN-γ production, resulting in a overall switch from Th1 to a protective Th2-type response (26). A couple of years later, Wasserman and colleagues observed, in the same model, that mice under IL-33 treatment were unable to increase the number of cells with regulatory properties as wild-type controls (32). They also observed a poor expression of ST2 on CD4+ T cells from sick mice, which may be a reason for the unresponsiveness to exogenous IL-33 (and the little increase of Tregs under this treatment). However, the lack of membrane-bound ST2 is explained by a mayor concentration of soluble ST2 (quantified by ELISA), which is a decoy receptor that avoids the downstream signaling when IL-33 is bound, preventing response to the cytokine (32). Altogether, this evidence suggests that the IL-33/ST2 axis is involved in the development of atherosclerosis, and proposes that a problem on IL-33 signaling may affect the ability of the organism to respond adequately to an unbalance of the organism homeostasis.

With respect to the transplantation field, in 2011, Brunner and collaborators showed that periodic IL-33 treatment of heart-transplanted mice resulted in an extended graft survival (33). This observation was associated to an increment in CD4+Foxp3+ Tregs population, which modulates the response to the graft. Also, the CD11b + Gr1int myeloid suppressor-derived cells (MDSCs) were augmented, contributing to the acceptance of the graft. Another interesting point made in this study is the complete change in the cytokine pattern: mice treated with IL-33 showed a decreased of IL-17A production, but an increase of IL-13, IL-5, and IL-10 production of *in vitro*-cultured, purified graft-infiltrating cells (33). With these data, the authors associated the improvement on graft survival with a Th2-type response, which favors the induction of regulatory cell populations such as Tregs and MDSCs.

In this same topic, our own group has seen an increment in CD4+FoxP3+ T cells in mice grafted with semi-allogeneic skin transplant and treated with IL-33, which is also related to a better state of the graft, a blockade in IFN-γ and IL-17 production and increased skin graft survival.

The information above shows that IL-33 participates in the induction of tolerance by targeting CD4+ T cells and some APC populations, orchestrating an immune response to favor an immunoregulatory status of the organism.

The data from all models (autoimmunity and transplantation) indicate that IL-33 is involved or may act at different stages of the immune response resulting (most likely) in a protective immunity for the organism.

### IL-33 and CD8+ T cells

The first role of IL-33 in immunity was linked to the innate-type response. But later, several reports showed that IL-33 is able to modulate adaptive immunity, mainly by acting directly on CD4+ T cells. Until 2009, the role of IL-33 on cytotoxic CD8+ T cells (Tc cells) was totally unknown. The first study elucidating a potential function of IL-33 on this T cell population was reported by Yang and collaborators. His work focused on the contribution of IL-33, in a TCR-dependent and -independent fashion, on human and murine CD8+ T cell biology *in vitro*. First, they performed gene arrays analysis on murine type-I Tc cells and found that IL-33 was one of the genes highly upregulated (34). Since IL-33 affects Th1, Th2, and Th17 differentially, the investigators stimulated Tc cells under type-1, -2, and -17 conditions, and measured ST2 mRNA. The results indicated that ST2 is up-regulated on Tc1 and Tc17 cells, but IL-4 inhibited ST2 mRNA on Tc2 cells. Interestingly, ST2 expression on Tc1 seems to be dependent on T-bet, as shown in experiments using *T-bet* KO CD8+ T cells. Conversely, exogenous IFN-γ did not alter ST2 expression neither at mRNA nor at the protein level (34). Considering that ST2 expression depends on TCR stimulation and T-bet, the authors evaluated the contribution of exogenous IL-33 on CD8+ T cell biology. According to the data reported above, IL-33 plus IL-12 upregulated IFN-γ, T-bet, and Blimp-1 expression on Tc1 cells, downregulating Eomes and IL-7R levels (T cell memory-associated molecules), without affecting the production of Granzymes or Perforins. Taking together, IL-33 and IL-12 enhance effector phenotype on CD8+ T cells (34). As in Th1 cells, these results are partially dependent on Gadd45b, a protein involved in CD4+ T cell arrest and differentiation (34). Another interesting report focused on identifying molecules involved in the induction of anti-viral responses. In this work, mice were infected with lymphocytic choriomeningitis virus (LCMV), and their splenocytes were harvested to analyze gene expression changes triggered by LCMV infection. From a panel of cytokines and other molecules, IFN-γ and IL-33 were the most up-regulated ones, in addition to ST2 (35). IL-33 contribution to anti-LCMV response was evaluated infecting *Il-33^−/−^*, *Il1rl1-Fc*, and *Il1rl1^−/−^*mice, resulting in an impairment of the cytotoxic T lymphocyte (CTL)-mediated response. In a similar approach, ST2-deficient animals infected with murine herpes virus 68 (MHV-68) presented elevated viral titers, which it was reversed when administering exogenous IL-33, suggesting that IL-33 signaling is necessary for virus clearance or an effective CTL response. This hypothesis was finally proved performing bone marrow chimera experiments, in which only wild-type CTLs were responsive to LCMV infection versus the ST2-deficient counterpart, given in part by a reduced survival and the lack of granzyme B and CD107a expression (important molecules involved in cytotoxicity) by the deficient cells (35). On the other hand, this experimental approach permitted to identify the non-hematopoietic compartment as the main source of IL-33 during viral infection. Finally, a very recent manuscript describes the role of IL-33 on tumor immunology. In this report, the investigators engineered two tumor cell lines to over-express (and secrete) IL-33. When injected into mice, the IL-33-producing tumor grew less, and the tumor infiltrating lymphocytes (TILs) were more abundant than in mice injected with control tumor cells. Moreover, when TILs were characterized, CD8+ T cells were enriched as in controls, but they displayed a more activated and differentiated phenotype given by elevated expression of the transcription factors T-bet and Eomes, higher IFN-γ secretion, and granzyme B production, demonstrating that IL-33 controls CD8+ T cells differentiation and effector function, which can be manipulated for anti-tumor therapy (36).

Taken together, the findings described above indicate that not only CD4+ T cells get triggered by IL-33 but also CD8+ T cells, supporting the idea that IL-33 may modulate T cell-dependent functions, which in turn can be reflected on the nature of the adaptive immune response.

## Future Perspectives

The information above clearly manifests the variety of roles that IL-33 exerts in the immune system, considering both innate and adaptive types of responses. To manipulate and translate IL-33 functions to treat disease and restore immune homeostasis or tolerance, we need to understand in a more detailed way the IL-33 participation during autoimmunity and graft rejection. Current studies using animal models show that IL-33 is present in the inflammatory response triggered during disease, but extensive studies are required using cells and tissue from patients. Recapitulating the roles of IL-33 in human settings can open new opportunities to design immunological therapies. Of our interest is the field of Tregs, where IL-33 positively impacts their biology, by mainly expanding or increasing their number *in vivo*. Is this response also observed on human Tregs? Are patient’s Tregs sensitive to IL-33 treatment? Is ST2 expression altered during disease? Are IL-33-expanded Tregs able to migrate to inflamed tissue? Many questions are still unsolved.

At present, there are some biological therapies that target cytokines for treating diseases; thus, a similar approach can be used for IL-33. Similar to IL-33, IL-2 therapy was sought to enrich for Tregs, since this cytokine acts as a growth factor for T cells. In an animal model of diabetes, low dose of IL-2 promoted Treg accumulation and function, and favored a switch in cytokine milieu given by a reduction on IFN-γ production. Interestingly, this low dose of IL-2 did not alter the frequency of other cell types (including effector T cells), but Treg number (47). In humans, IL-2 administration has also showed benefits, such as an increase in functional Treg numbers and a reduction in inflammatory signs [GVHD and vasculitis patients (48, 49)]. Alternatively, an antibody anti-TNFα was designed to treat RA. This cytokine is normally produced in response to local injury; however, it drives an exacerbated inflammation producing tissue damage when secreted in excess. In RA, TNF-α is present in the synovial fluid from patients. At date, there are five biological inhibitors approved by the FDA, three of them are monoclonal antibodies (infliximab, adalimumab, and golimumab); Etanercept is a fusion protein of two TNFR2 receptor extracellular domains plus the Fc fragment of human immunoglobulin 1, and Certolizumab is a humanized Fab fragment conjugated to polyethylene glycol (50). Although the patient life quality improves, they are not an effective treatment due to a loss of effectiveness or severe secondary effects, such as higher risk to serious infections or lymphoma (50). Other treatments targeting cytokines or their receptors include the anti-IL-6R antibody. This approach was developed considering the high amounts of IL-6 in the synovial fluid of patients. The receptor for this cytokine, IL-6Rα is found in a wide range of cells, including T cells, monocytes, activated B cells, neutrophils, and hepatocytes (51). Recently, a monoclonal treatment has been proved (Tocilizumab), which is capable to block IL-6R, producing an improvement in the symptoms. The main problems with this therapy are the secondary effects associated with its use. Since IL-6 is a very pleiotropic cytokine, many tissues and/or organs get affected, including elevated propensity to infections, malignant tumors, changes in the blood and lipid profile, hepatic problems, and cardiovascular danger (52).

Until date, specificity is the main obstacle for the application of this type of approaches, considering that (all) cells expressing or producing the cytokine and its receptor could get triggered by the administered drug. Therefore, when thinking in the possibility to use IL-33 for human treatment, we need to be specific regarding its target, because of its wide effects on cell types and, consequently, in the microenvironment. This phenomenon is related to the signaling pathways triggered during the encounter between the cytokine and its receptor. For this reason, a better knowledge on IL-33 signaling and its interaction with others molecules are really necessary. Based in the different responses observed in a same animal model for diseases, we could think that dosage, timing, microenvironment, and state of the disease are key variables that may affect the final outcome of IL-33 treatment; thus, determining when these factors are aligned to favor a polarization to Tregs (for autoimmunity or block graft rejection) would be useful to act positively in the patient.

Lastly, the administration method is not less important. For delivery of sensitive molecules, such as cytokines, an interesting solution is the use of novel technologies. Nanomedicine supports the control, repair, and improvement of the human biological system working from the molecular level, and it has been proposed as a possible solution for the controlled release of a desired molecule. It consists in establishing nanostructures to carry a drug to an affected area, encapsulating, and releasing it at its destination under specific conditions (53). This dynamic mechanism is called nanotherapy and it is characterized by several benefits including improvement in disease detection and good rate of absorption and treatment of patients. Among the nanostructures designed, we can find nanoparticles, nanocapsules, dendrimers, liposomes, micelles, nanotubes, and microgeles, each of one with characteristic properties. Applying the above to IL-33 may be a great opportunity to create a therapy through specific targeting leading to an effective cure to restore immune tolerance (54).

In summary, there is still a lot to know and understand about IL-33; however, the results shown to date gives us positive expectations on a new therapy involving this cytokine.

## Conflict of Interest Statement

The authors declare that the research was conducted in the absence of any commercial or financial relationships that could be construed as a potential conflict of interest.
